# Development of a Stereotaxic Device for Low Impact Implantation of Neural Constructs or Pieces of Neural Tissues into the Mammalian Brain

**DOI:** 10.1155/2014/651236

**Published:** 2014-01-23

**Authors:** Andrzej Jozwiak, Yiwen Liu, Ying Yang, Monte A. Gates

**Affiliations:** ^1^Institute for Science and Technology in Medicine (Hartshill Campus), Medical School, Keele University, Thornburrow Drive, Hartshill, Stoke-on-Trent ST4 7QB, UK; ^2^Institute for Science and Technology in Medicine, School of Life Sciences, Keele University, Keele ST5 5BG, UK

## Abstract

Implanting pieces of tissue or scaffolding material into the mammalian central nervous system (CNS) is wrought with difficulties surrounding the size of tools needed to conduct such implants and the ability to maintain the orientation and integrity of the constructs during and after their transplantation. Here, novel technology has been developed that allows for the implantation of neural constructs or intact pieces of neural tissue into the CNS with low trauma. By “laying out” (instead of forcibly expelling) the implantable material from a thin walled glass capillary, this technology has the potential to enhance neural transplantation procedures by reducing trauma to the host brain during implantation and allowing for the implantation of engineered/dissected tissues or constructs in such a way that their orientation and integrity are maintained in the host. Such technology may be useful for treating various CNS disorders which require the reestablishment of point-to-point contacts (e.g., Parkinson's disease) across the adult CNS, an environment which is not normally permissive to axonal growth.

## 1. Introduction

Destruction or damage to neural circuits in the adult mammalian central nervous system (CNS) is notoriously difficult to repair. Indigenous cells rarely regenerate across the adult CNS, and neural circuitry reconstruction is made difficult by the fact that the adult CNS expresses molecules, that inhibit axonal growth, and/or fails to express precise gradients of growth-promoting cues that would stimulate/direct axonal growth [1–4]. Transplantation of immature cells has been considered a potential therapeutic strategy for the damaged adult brain and spinal cord, and there is currently sustained interest in the generation of stem cell lines that could be used to treat certain CNS injuries or disorders. Parkinson's (PD) and Huntington's (HD) diseases are two such instances where a substantial amount of research is being conducted to discover the potential for structural repair of neuronal circuits (via cellular transplants) when effective alternative therapies (e.g., pharmacological therapy) become ineffective [5–7].

In the case of PD, where the progressive and selective loss of dopaminergic neurons within the substantia nigra (SN) leads to dopaminergic denervation of the striatum, one possible solution has been to transplant embryonic nigral cells into the host substantia nigra (SN), in an attempt to rewire nigrostriatal circuit. Past studies have shown that some fetal nigral cells transplanted in this way can grow a limited number of axonal projections towards the striatum, but most are generally incapable of growing over the distance required to establish functional connections in the striatum in the adult brain [8–10]. Though cells from the foetal brain (on their own) are rarely able to reinnervate their relevant target unless they are placed close to, or within, the target region [11], recent work has shown a limited potential for homotopically transplanted nigral cells to functionally be connected with the striatum in the rodent model of Parkinson's disease when stimulated by certain growth factors within the transplant and/or target region [12, 13].

However, the most common strategy for circuitry repair in the CNS has been to implant dissociated cells directly into target sites (i.e., the striatum in case of PD; [14–19]). Recent research has shown that pieces of fetal nigral tissue placed in the striatum of 6-OHDA lesioned rats offer greater cell survival and predictability of graft function (in comparison to dissociated nigral cells) in the animal model of Parkinson's disease [20]. However, such heterotopic transplants, although capable of alleviating symptoms that benefit from neurotransmitter supplementation, do not re-establish the natural homeostatic regulation of neural activity in the brain and dramatically limit the cell/circuitry replacement strategy to practically only PD. Even in the case of PD, such heterotopically transplanted cells release dopamine in an unregulated way and may be responsible for various untoward complications associated with the current cell transplantation strategy (e.g., the dyskinesias observed in Parkinsonian patients receiving foetal cells transplants; [21–23]).

At present, it is thought that the efficacy of cell replacement in the CNS would be greatly enhanced if a means to fully reestablish degenerated or disrupted pathways (e.g., nigrostriatal) could be developed. In this context, current efforts focus on overcoming the effects of potent neurite growth inhibitory factors in the adult CNS [24] or providing the growth promoting cues to immature neurons [12, 13] before, during, or after standard dissociated cell transplantation. Other approaches involve improving the axonal growth of homotopically transplanted neurons by creating a growth supporting bridge, via cografting various cell types along the nigrostriatal axis [25], or by stimulation of the localised release of GDNF or the excitatory amino acid kainic acid, [12, 26–28]. These studies have reported some success, along with recent work on monkeys revealing that grafted dopaminergic neurons can extend neurites to a distant target in nonhuman primates. This indicates that homotopically grafted neurons can be reconnected with distal regions of the CNS if a correct strategy can be found that allows transplanted cells/tissue to interact with the sites they are to be connected with [29].

However, identifying all the growth promoting and inhibitory cues that need to be balanced to bring about extensive axonal growth in the adult host brain after homotopic transplantation is a daunting task. Another strategy might be to preconstruct neural circuits *in vitro* (“neural wires”), or dissect out immature circuits, before their implantation to the brain, and implant them, intact, in their normal orientation, *in vivo*. Such implantation of constructed “neural wires” or an intact neural circuit would by-pass the untoward effects of the host tissue (such as the expression of inhibitory molecules within the adult host) that limit axonal growth [30] by reconnecting two regions of the adult brain at the moment of implantation. Realisation of such concept, however, requires developing new methods for the formation of neural constructs *ex vivo* and, in particular, solving technical difficulties related to implanting such a construct or piece of tissue with orientation and integrity.

**Figure 1:**
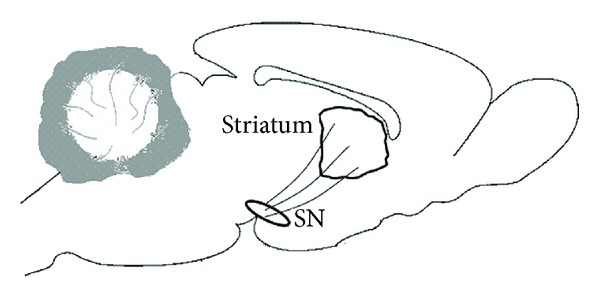
A midsagittal view of the rat brain highlighting pathway between the substantia nigra (SN) and striatum. This nigrostriatal circuit was used here as a model system to test the potential to place a neural construct along the length of its pathway, as it is contained within the CNS, has certain clinical relevance (i.e., in research on Parkinson's disease), and is a well-defined, short circuit of the brain.

Here, a method has been developed whereby scaffolds may be formed in a tubular (glass) capillary that can be used to encase and grow neural tissue and then subsequently used as part of an implantation device to place the scaffold/tissue construct directly into the brain (Figure 1). These multiple capabilities are enabled by the scaffolds being prepared within an implantation needle that can both support the growth of cells or tissue *in vitro* before use and protect the physical integrity of the preconstructed neural tissue (“wires”) during the implantation procedure. As a prelude to the use of the device on living animals, the implantation technique and implantation device are tested here for their potential to precisely deposit constructs at a desired location within the (postmortem) brain while maintaining the spatial orientation of the implanted tissue. The manifold technical aspects of the implementation concept are also demonstrated through the use of a “phantom system” which mimics implantation to the mammalian CNS and allows for real-time viewing of the implantation process. In general, this technology provides a tool for, and facilitates experimentation in, research directed at implanting engineered tissues or constructs into the CNS.

## 2. Materials and Methods

### 2.1. Scaffolds Preparation

Scaffolds were prepared directly within the tip of an implantation needle made of glass (0.5 mm external diameter and 0.4 mm internal diameter) fitted with a Luer connector (Hilgenberg GmbH, Germany). A 5 mm long porous wall scaffold was formed by the phase inversion method from a poly(lactide-co-glycolide) (PLGA) (Sigma UK) solution (35% in DMSO). Briefly, the polymer solution was pulled into the needle up to 5 mm from the tip by a syringe and then pushed out to create a thin film that was immediately coagulated in distilled water. Following the coagulation step, the needle was extensively rinsed in water and subsequently used for growth and implantation procedures. A dense scaffold can be prepared in a similar way using a PLGA solution in chloroform (35%), where the water coagulation step is replaced by simple evaporation of the solvent. The needle with the scaffold is sterilised via UV radiation and can be stored for later use or used *in vitro *for growing neural cells/tissues within the scaffold to form neural circuits (biological “wires”).

### 2.2. Implantation Device

#### 2.2.1. Supplementary Tubular Plunger

**Figure 2:**
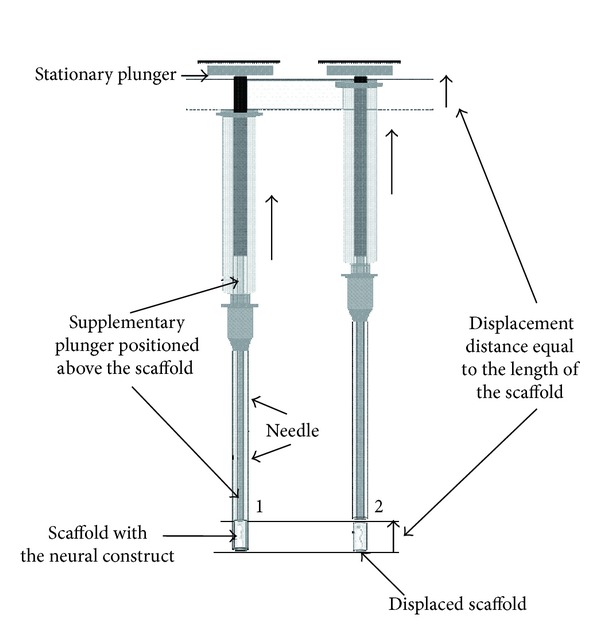
The principle of the low force displacement and deposition of the construct using the supplementary plunger between the plunger of syringe and the construct to be implanted. While both plungers stay in the fixed position (left), the syringe with the needle moves upwards (right), displacing the construct from the needle and depositing it within the space formed by the retracting needle.

Following a culture phase, or after placing a piece of dissected tissue within the needle, the needle containing the construct (or tissue/cells within the scaffold) at the tip remains filled with culture media. To enable the controlled displacement of the neural construct from the needle, a supplementary, tubular plunger operating within the needle, is utilized. Because the plunger is hollow, the media filling the needle can exit upwards through the bore of the supplementary plunger while it is being inserted into the implantation needle. This prevents the development of any downward force on the neural construct that would dislodge it from the needle or distort it while the implantation device is being assembled. The plunger is a glass capillary matching the internal diameter of the glass needle, yet it is slightly longer than the implantation needle itself. For the implantation procedure, the needle with the supplementary plunger is fitted onto a 10 µL Hamilton syringe via a three-way connector with a valve, in such way that the supplementary plunger is positioned precisely against the plunger of the syringe at the proximal edge of the scaffold (located in the distal tip of the capillary). Thus, the supplementary plunger extends the action of the syringe plunger to the full length of the implantation needle (Figure 2). The three-way connector with the valve in the open position allows for fitting the needle onto the syringe without creating pressure which could push the construct out of the needle. Before the implantation surgery, the valve is closed.

#### 2.2.2. Implantation Mechanism

**Figure 3:**
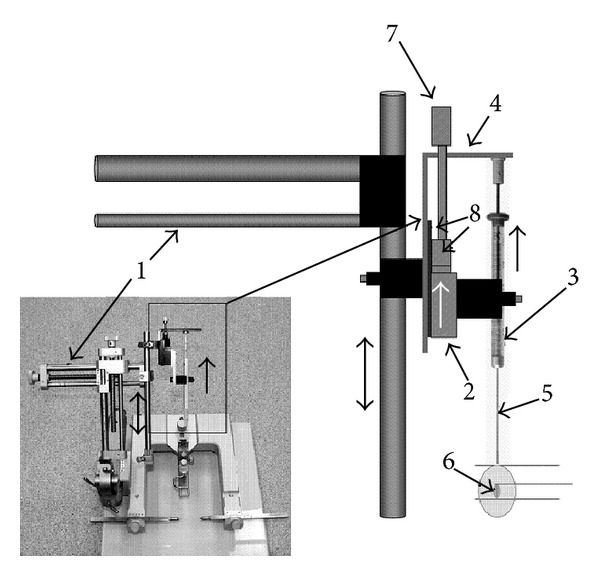
Diagram of the implantation device attached to a stereotaxic frame. An implantation accessory bracket (4) with the stationary section (8) of the translation stage is fitted to the arm of the stereotactic frame (1). A movable section of the translation stage (2) allows for retracting of the syringe (3) by turning the micrometric screw (7). After insertion into the brain, using the stereotaxic frame, the needle (5) is retracted jointly with the syringe, while the plunger, being fixed to the accessory bracket (4) is stationary together with the supplementary plunger within the needle and the scaffold itself. As a result, scaffold (6) is displaced into a cavity formed by the retracting needle. Photograph (lower left) shows the implantation device mounted on the stereotactic frame.

An implantation device has been designed as an accessory to be mounted onto a conventional stereotactic frame. The device comprises two main sections (Figure 3): a stationary and a movable section. The stationary section contains an accessory bracket coupled to the base of a precise translation stage mounted to the arm of a stereotactic frame. The movable section is composed of the movable segment of the translation stage with the 10 µL Hamilton syringe/plunger assembly (detailed above) clamped onto it. The desired position of the implantation accessory (and the implantation needle tip) is attained by the relevant settings of the stereotaxic frame, while the implantation accessory itself enables precise (i.e., with an accuracy of within 0.1 mm) movements of the implantation needle via the movements of the translation stage. Since the plunger of the Hamilton syringe is fixed to the accessory bracket, when the syringe is moved upwards (by turning of the micrometric screw of the implantation device), the plunger of syringe and the supplementary plunger remain stationary. This allows for the withdrawal of the needle and the displacement of the construct to occur at the same rate (“laying it out”), as the supplementary plunger keeps the implant stationary as the needle is raised, leaving it (the implant) *in situ* in the host tissue as the needle is removed.

### 2.3. Testing the Implantation Technique Using a Brain Phantom

**Figure 4:**
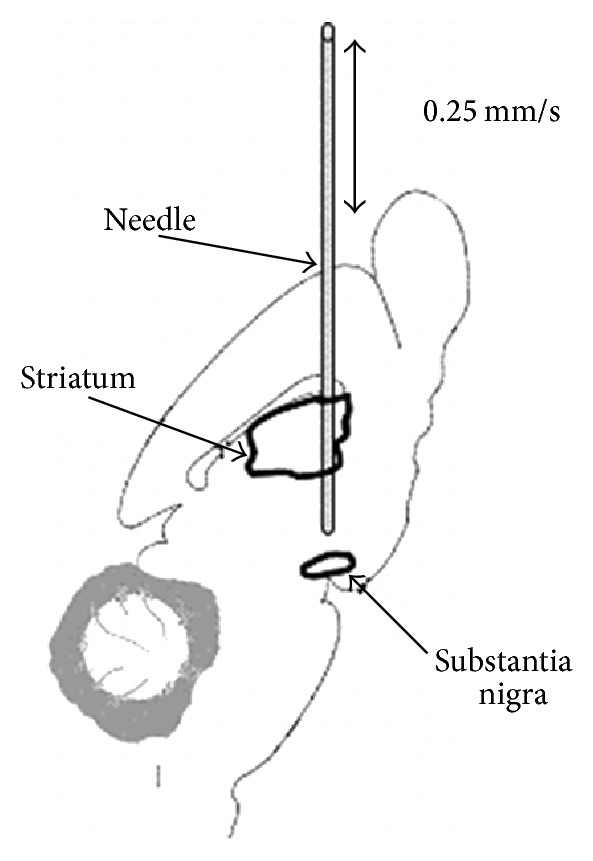
Schematic illustration of the trajectory followed by the needle used to test the mechanical resistance of the rat brain. A fresh, postmortem rat brain was affixed to an ElectroForce device and a probe lowered at 0.25 mm/s to measure the mechanical resistance along the full length of the nigrostriatal pathway.

The procedure of displacing a circuitry construct from the needle and testing its precision was tested using a phantom-gelatine gel (as an artificial substitute of brain tissue) and an empty scaffold. This allowed for the real-time viewing of the displacement process and enabled a direct observation of the preservation of the construct's integrity during implantation procedures. To do this, it was first necessary to establish the force of mechanical resistance of the gel so that it could be set to mimic relevant biological tissue. This was established using the ElectroForce 3200 Series test instrument to measure the friction placed on a needle during its insertion into a rat's brain. Briefly, a freshly isolated rat brain was positioned on the instrument stage and a stainless steel flat tipped (0.5 mm in diameter) needle was fitted to the instrument and driven (at the rate of 0.25 mm/s) into the brain along a line between the striatum and the SN (Figure 4). To determine a concentration of gel that matched the tension produced by the rodent brain (a “phantom gel”), gelatine gels, at concentrations 1%, 2%, and 3%, were prepared in disposable, transparent spectrometer vials and similarly tested at the same instrument settings to identify a gel concentration with similar mechanical resistance to that shown with the fresh rat specimen. Subsequently, the displacement of the scaffold was examined within a concentrated phantom gel placed in disposable cuvettes using the implantation device mounted on a stereotaxic frame. Displacement of the scaffold from the implantation needle was recorded using a USB digital microscope.

### 2.4. Simulated Rat Surgery

**Figure 5:**
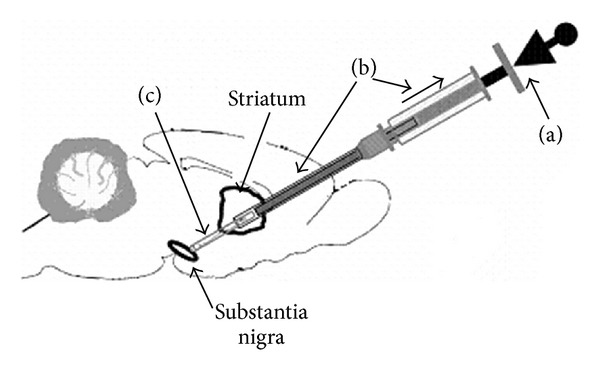
A schematic diagram showing the trajectory of the implantation device containing a scaffold construct along the nigrostriatal pathway. Note, for illustration purposes, the implantation device is shown here much larger than it is in relation to the rodent brain. (a) illustrates how the plunger of the Hamilton syringe is held in place as the syringe and needle (b) are moved upwards, “laying out” the construct (c) *in vivo* as the glass capillary is removed.

To test that the device was capable of precisely implanting a construct into the CNS, fresh, postmortem rats were placed in stereotaxic frame and the implantation needle with the scaffold containing small pieces of tissue was inserted along the coordinates, 5.5 mm anterior to bregma, 2.5 mm lateral to the midline, and 11.5 mm below the dura, at the angle 55° from the vertical (Figure 5). After implantation, the brain was removed, fixed in 4.0% paraformaldehyde in phosphate-buffered saline, and subsequently cryosectioned to view the implant placement.

## 3. Results and Discussion

### 3.1. Scaffolds

**Figure 6:**
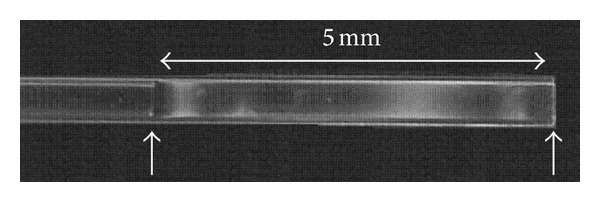
High magnification image showing a scaffold formed within the needle tip. Arrows point to the both proximal (left) and distal (right) ends of the scaffold. Opaque areas indicate thicker sections of the scaffold wall specific for the manual manufacturing process, which improve the visibility of the scaffold during the tests.

Scaffolds were prepared by the phase inversion method directly within the glass implantation needle. Considering the approximate distance between the SN and the striatum, the capillary scaffolds for the nigrostriatal circuit were 5 mm long, with an external diameter of 0.4 mm and wall thickness of 15–30 micrometers (Figure 6). The translucent scaffold has an opaque ring at the proximal end intentionally shaped from a thicker layer of polymer solution to enlarge the contact area for the supplementary plunger to hold fast the scaffold during implantation. Other opaque areas of the scaffold, which result from the manual film formation procedure, are helpful in observing of the scaffold during the procedure of filling it with the cellular material.

The phase inversion technique employed for the scaffolds preparation allows for a relatively easy regulation of the structure of the scaffold wall, using established membrane technology methods. The structure of the scaffolds can be suitably modified to provide selective permeability of macromolecules, as well as different biodegradability and surface chemistry [31, 32]. In fact, most of the established biocompatible medical polymers can be processed into thin walled tubular scaffolds using the phase inversion technique, offering a wide range of options regarding the scaffold properties and functions. When a biodegradable scaffold is used *in vivo*, it may conveniently allow for the controlled release of trophic factors that may provide additional protection and stimulation of the implanted neural circuit's growth, as well as preventing further neural degeneration of the host brain [33–37]. It may also serve as a source for factors that stimulate the growth of the neurons in the *in vitro* cultivation phase. Finally, the scaffold can protect the spatial structure of the grown neuronal construct during implantation and also partially shield the newly implanted neural circuits from the unfavourable host environment during the early period of reintegration.

An important advantage of preparing the scaffold within the implantation needle using this method is that larger as well as smaller diameter needles can be used as necessary, and suitable scaffolds can be prepared relatively easily. This provides additional flexibility in the choice of the size of the implanted construct (and the implantation needle) and combats the known negative effects that the size of some transplantation needles/cannula may have on the survival of grafted cells [38]. Additionally, the size of the implant may also be adapted according to other particular requirements, like the size of the brain and the neural tract distance, or the required volume of the neural circuit.

### 3.2. Implantation Method

Preparation of the tubular scaffolds directly within the implantation needle offers several practical advantages. It eliminates technical difficulties associated with loading the scaffold or neural construct into an implantation tool (needle) and provides a way for forming neural circuits/“wires” in a way that secures their integrity and orientation during implantation. Furthermore, using the implantation needle as a bioreactor provides an “all in one” implantation device, easily adapted for standard cell culture equipment (i.e., petri dish, with media, and an incubator). If applied experimentally (or possibly in the clinical setting), such a technique would provide the additional benefit of growing several neural constructs in parallel and allow for a quality period as well as the added flexibility in the time for surgical implantation.

However, this approach requires a specific technique to resolve technical difficulties related to the preparation of the scaffold and the displacement of the construct from the implantation needle while protecting the integrity of the preconstructed neural tissue (“wires”). To address these issues, a supplementary tubular plunger operating within the implantation needle has been devised (Figure 2). The tubular plunger can be inserted into the implantation needle without creating a flow of culture media through the needle tip (and the construct/tissue it contains) as the media can escape back up the tubular plunger. The needle, with the supplementary plunger positioned precisely at the edge of the scaffold, is then fitted onto the Hamilton syringe, in such a way that the supplementary plunger is positioned exactly between the edge of the scaffold (construct) and the plunger of syringe (Figure 3). Thus the action of the syringe's plunger is extended to the full length of the implantation needle by the tubular plunger and is controlled by the displacement mechanism (as described above).

The great advantage of this displacement mechanism is that it allows for the direct control of the physical deposition of the construct in the recipient tissue, as the distance moved by the plunger of the Hamilton syringe is directly related to the amount of displacement occurring for the construct at the tip of the needle (Figures 2, 3, and 5). During implantation surgery, the implantation needle is positioned within a chosen area of the brain using the stereotactic frame (Figure 5). Subsequently, using the implantation accessory device, the needle is retracted for the required distance equal to the length of the scaffold, while the scaffold, being blocked by the both stationary plungers, stays in its place. In this way, the scaffold containing the neural circuit can be deposited (“laid out”) exactly into the space vacated by the retracting needle (Figures 3 and 5). After the construct is displaced into its target position, the needle is removed from the brain using the stereotactic frame controls. This technique allows for deposition of the neural construct in a precise location, with a particular orientation, while minimising force on the host tissue and without disturbing the spatial organization of the implanted material. In principle, this implantation device can be used in other configurations, (e.g., without the supplementary plunger) where implanted material (e.g., dissociated cells or pieces of tissue) can be displaced from the needle using culture media, mineral oil, or by other suitable pressure transducing media.

### 3.3. Phantom System

**Figure 7:**
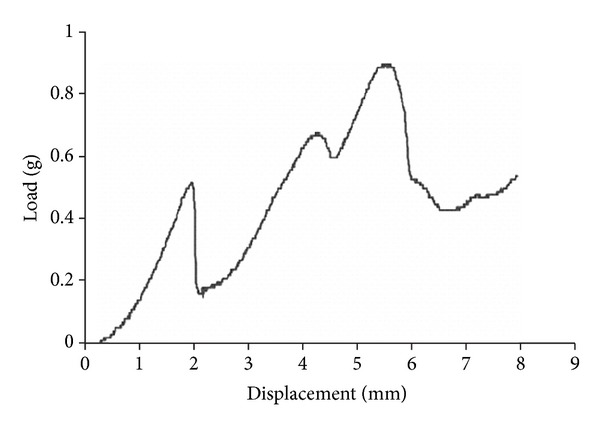
Characteristic friction profiles measured from the ElectroForce needle inserted into the rat brain along the approximate path linking the striatum to substantia nigra. Note the range of mechanical resistance varies as the probe is lowered. This is due to the needle interacting with different densities of tissues (i.e., grey and white matter) as it probes through the brain.

**Figure 8:**
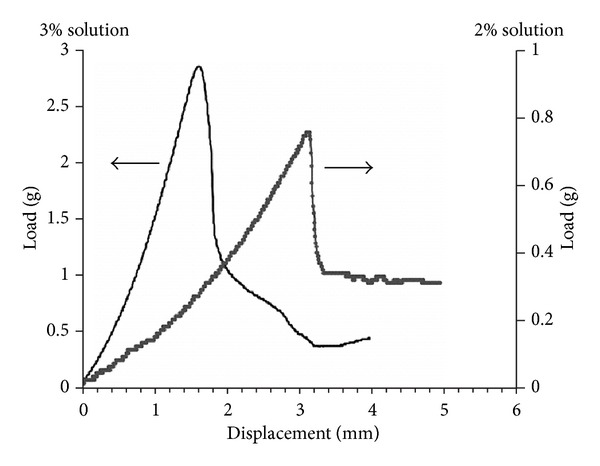
Characteristic friction profiles measured from the ElectroForce needle inserted into the brain phantom made from 3% (left scale) and 2% (right scale) gelatine solution. Note that the range of mechanical resistance is much smoother (in comparison to the rat brain) and that the resistance shown by 2% gels more closely matches those seen with the rat brain specimen (Figure 7).

The brain phantom, made of a transparent gelatine gel, greatly facilitated the testing of the implantation procedures and the implantation device, allowing for the continuous observation of the scaffold/construct displacement in real-time. In order to prepare a gel with the appropriate mechanical properties relative to brain tissues, the resistance of real brain tissue was first measured as a reference. The measured friction profile for the inserted needle revealed the mechanical resistance of the rat brain to the implantation needle (Figure 7). The gelatine gels produced friction profiles characteristic for homogenous structures, with only one peak representing puncture of the denser top layer. The 1% gelatine proved to be too soft to produce any measurable resistance. The 3% gelatine created much higher measured loads, while the 2% gelatine solution friction profile remained within the range of that for that seen with the rat brain (Figures 7 and 8) and was chosen as closely imitating the resistances met within the brain tissues.

### 3.4. Testing of the Scaffold Displacement with the Brain Phantom

**Figure 9:**
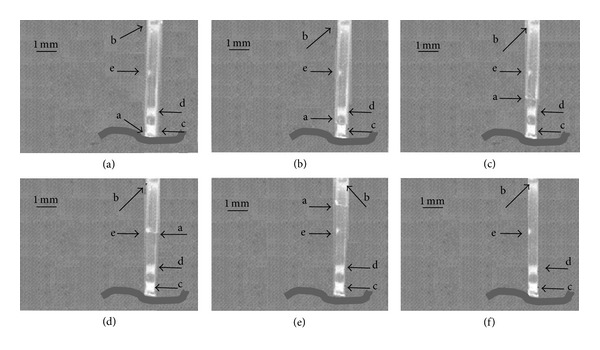
Time-lapse images (1–6) illustrating precise deposition of the scaffold at a marked position (dark line at bottom of image) within the brain phantom. (a) The tip of the needle; (b) the top end of the scaffold; (c), (d), and (e) marking points on the scaffold indicating lack of deformations during displacement. Note that as the needle tip (a) is raised, the scaffold is left stationary and in the position it was originally lowered to within the gelatine gel.

The brain phantom system allowed for uncomplicated verification of the implantation device function. Tests confirmed expected high accuracy of the deposition of the scaffold without distortion of the implanted construct (Figure 9). Time-lapse images show that the marking points on the scaffold maintained their original positions during the whole displacement procedure and no deformation of the scaffold was observed. The scaffold itself rested precisely at the target location after implantation was complete.

### 3.5. Implantation into the Rodent Brain

**Figure 10:**
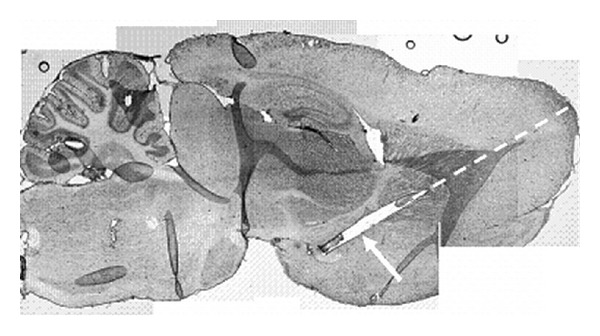
Montage of a sagittal section of the brain showing a scaffold (white arrow) linking the substantia nigra to the striatum (5.5 mm anterior to bregma, 2.5 mm lateral to the midline and 11.5 mm below dura, 55° from the vertical). Dashed line indicates insertion trajectory and the arrow points to the scaffold. Note the small piece of tissue remaining in the caudal tip of the scaffold construct.

A simulated surgery experiment was performed on a postmortem rat to confirm functionality of the implantation device in neural tissue and to establish the implantation procedure's use for *in vivo* studies (Figure 10). As shown in Figure 8, the implant was laid out along the chosen stereotaxic coordinates, and the scaffold's structure remained perfectly intact after implantation procedures. Visible fragments of the tissue at the end of the scaffold show pieces of the tissue inserted into the scaffold before implantation. Their position was not affected by the implantation procedure, as they were positioned at the rostral end of the midbrain substantia nigra.

## 4. Conclusions

Here is presented a novel, integrated technology that enables implantation of neuronal tissues (“wires”) and/or constructs placed within a tubular (capillary) scaffold, for the restoration of damaged neural connections between two nuclei in the adult CNS. This technology was developed in response to the current interest in establishing functional neural constructs along broken neural tracts; as a potential means to alleviate various undesired side effects that may be related to the unregulated activity of heterotopically placed cell transplants and improve chances for the restoring neural circuitry. Firstly, the technology outlines a technique for preparing scaffolds within an implantation needle that can also be used as scalable culture environment for the *in vitro* growth of neural tissue (circuits). Such an approach further benefits from a straightforward preparation and growth of the constructs using standard cell culture equipment and the possibility of additional screening prior to implantation.

Secondly, the implantation of scaffolds containing neural circuits/tissue (or neural constructs) is made possible by an implantation technique and device that offers a simple, one-step, one-time insertion procedure where the implanted construct is precisely “laid” into a space formed by the retracting needle. Such precise deposition and orientation of the implanted material are particularly important when the implantation is aimed at small target structures like the substantia nigra in rats or mice. The implantation technique and device generally allow for the implantation of various types of scaffolds or implants, including soft tissue explants or hydrogels, lending great flexibility to the technology's application. In general, the concept of preconstructing neural circuits *in vitro* together with the associated implantation technology is naturally applicable for advances being made in stem cells research [39–43] and may begin to offer a new avenue for cell replacement strategies that aim to treat other CNS disorders which require the reestablishment of point-to-point contacts. Recently, hydrogels have been widely used as carriers for growth factors and cells [44, 45], but delivery of the constructs remains a difficulty [46]. The technology highlighted here would enable one to grow neural tissue/cells within a medium (such as a hydrogel) within a scaffold and displace this construct *in vivo* in a way that would not disturb the (pre)organisation/growth of the construct. Indeed, it seems possible that a wide range of tissues or cells could benefit from a clear, unperturbed route between nuclei in the CNS and that constructs (either with formed “neural wires”, or as bridges that facilitate growth) could improve the possibility of connecting distal parts of the CNS by offering such a route at the moment of implantation.
